# Temperature Controls Onset and Period of NF-*κ*B Oscillations and can Lead to Chaotic Dynamics

**DOI:** 10.3389/fcell.2022.910738

**Published:** 2022-06-20

**Authors:** Mathias Heltberg, Mads von Borries, Poul Martin Bendix, Lene B. Oddershede, Mogens H. Jensen

**Affiliations:** Niels Bohr Institute, University of Copenhagen, Copenhagen, Denmark

**Keywords:** dynamical systems, oscillations, gene regulation, controllability, chaos, transcription factors, NF-kB

## Abstract

The transcription factor NF-*κ*B plays a vital role in the control of the immune system, and following stimulation with TNF-α its nuclear concentration shows oscillatory behaviour. How environmental factors, in particular temperature, can control the oscillations and thereby affect gene stimulation is still remains to be resolved question. In this work, we reveal that the period of the oscillations decreases with increasing temperature. We investigate this using a mathematical model, and by applying results from statistical physics, we introduce temperature dependency to all rates, resulting in a remarkable correspondence between model and experiments. Our model predicts how temperature affects downstream protein production and find a crossover, where high affinity genes upregulates at high temperatures. Finally, we show how or that oscillatory temperatures can entrain NF-*κ*B oscillations and lead to chaotic dynamics presenting a simple path to chaotic conditions in cellular biology.

## 1 Introduction

The fine-tuned regulation of protein production is fundamental to all living organisms. This production is complex and includes a number of components, but central to the stimulation of genes is the concentration of transcription factors inside the nucleus. In the past two decades, it has been revealed that the nuclear concentration of a number of central transcription factors, can be highly dynamic and it is expected that this dynamics might be an important element in the complex gene regulation of cells. One example of such a transcription factor, is the p53 tumour suppressor that has a period of ≈5 h (Lahav et al., 2004; Chen et al., 2016; Heltberg et al., 2019a) and another, which is the scope of the present work, is NF-*κ*B, which oscillates with a period ≈1.5 h and has been shown to control the production of a number of proteins related to the immune response (Gonze et al., 2002; Hoffmann et al., 2002; Nelson et al., 2004; Krishna et al., 2006; Levine et al., 2013; Kellogg and Tay, 2015). It is at present debated what the functional role (if any) of these oscillations is, but it seems certain that the downstream genes are affected by this dynamics (Hoffmann et al., 2002; Nelson et al., 2004; Mengel et al., 2010; Tay et al., 2010; Heltberg et al., 2021).

The NF-*κ*B signaling pathway is one of the most essential signaling pathways in eukaryotic cells. Among other functions, it has a role in cancer, inflammation, ageing, and in the immune defence, and moreover, it is serving as a general stress response (Wang and Tarbell, 1995; Mercurio and Manning, 1999; Hoffmann et al., 2002; Nelson et al., 2004; Courtois and Gilmore, 2006; O’Dea and Hoffmann, 2009; Sugioka et al., 2010; Kellogg and Tay, 2015; Liu et al., 2015; Mitchell et al., 2016; Heltberg et al., 2021). Since the regulation of temperature is also predicted to be a fundamental part of especially the immune response, we were interested in studying the interplay between oscillatory NF-*κ*B and variations in temperature. A heat shock protein-dependent mechanism has been proposed as a link between the NF-*κ*B signaling pathway and heat (Liu et al., 2015), and recently a model introducing a delay of the A20 signaling protein has been suggested (Harper et al., 2018), but we wanted to test this further, and establish the results for different levels of TNFα induction. Furthermore, little is known about the mechanisms of how the affected NF-*κ*B oscillations could affect downstream protein production which we also set out to investigate in this study.

The theory of how reaction rates were affected by temperature, was pioneered by the work of the polish physicist Marian Smoluchowski (Smoluchowski, 1916), who calculated the reaction rate between two spherical particles diffusing in a potential. Even though this theory is more than 100 years old, and is a part of every physics curriculum, it has rarely been applied to systems that show dynamical behaviour such as limit cycles.

In this paper, in parallel to the experimental investigation, we apply the theory of temperature dependent reactions, to predict how the oscillatory dynamics of the transcription factor NF-*κ*B is affected. In the experiments, we use both single and double additions of the ligand TNF-α as well as applying this through a custom made flow chamber thereby achieving a constant concentration in TNF-α, as opposed to a declining concentration as is the case if only added once [as done, e.g., in Ref. (Harper et al., 2018)] The latter condition enables us to detect clear oscillations in the nuclear concentration of TNF-α, and we succeed in varying the temperature from 32*°C* to 41.5*°C* in the flow chamber, where oscillations are still maintained. Through the analysis of these data we find a clear dependency of the period on the external temperature, so increasing the temperature leads to faster oscillations. By using a well-tested model of the dynamics of NF-*κ*B, we find a striking correspondence between the model and the experimental findings and our model additionally predicts that lowering the temperature can lower the threshold level of the Hopf Bifurcation which defines the onset of oscillations in NF-*κ*B. Furthermore we use the model to predict how the change in temperature might lead to a significantly different downstream production level. Finally, we simulate how an oscillatory temperature can affect the NF-*κ*B dynamics and we find, that this can lead to entrainment even for small temperature oscillations. Interestingly, for amplitudes of $\approx 2.5^{o}$ C we find a chaotic transition that we surmise can be used to study the effect of complex dynamics on transcription factors in the future.

## 2 Results

### 2.1 Experimental Set-Up and Quantification of Nuclear NF-*κ*B Concentration Dynamics at Different Temperatures

We investigate how temperature affects oscillations of NF-*κ*B in mouse embryonic fibroblast (MEF) cells, through three experiments:

• TNF-α was added once to the MEF cells,
• TNF-α was added twice with 40 min in between
• MEF cells were exposed to a constant concentration of TNF-α in a flow chamber.

Each type of experiment was conducted at 32.0°C, 34.5°C, 37.0°C, and 39.5°C while a temperature of 41.5°C was additinally used in the flow experiments. The sample was imaged in a fluorescence time-lapse microscope, where DsRed-labeled p65 would give information about the relative nuclear to cytoplasmic concentration of NF-*κ*B in the MEF cells.

In the single addition experiments, TNF-α was added to the MEF cells 2 min prior to initiation of the experiments and the concentration was increased from 0 ng TNF-α/ml to 10 ng TNF-α/ml. At t = 0 min the fluorescence time-lapse microscope would start imaging at 10–12 different locations every 10 min. Experiments were terminated after 20–48 h. In the double addition experiments, TNF-α was added to the MEF cells increasing the concentration from 0 ng TNF-α/ml to 10 ng TNF-α/ml 2 min prior to initiation of the experiments, and then after ≈38 min the TNF-α concentration was increased to 18 ng TNF-α/ml. These experiments were also terminated after 20–48 h. The reason we systematically tested different doses in the single- and double addition experiments, was to map out the dependency (if any) on TNF-α addition, and by testing it though different protocols, also through the constant flow experiments, is to explore the robustness and origin of the oscillations. In the flow experiments, the TNF-α concentration converged towards a value of 1 ng/ml. These experiments were terminated between 40 h and 7 days after initialization (See Methods for details).

**FIGURE 1:**
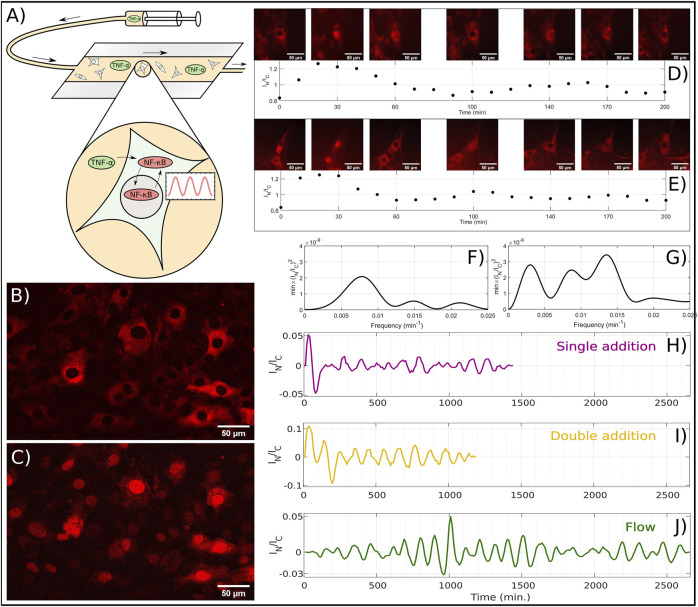
Experiments on NF-*κ*B oscillations conducted on mouse embryonic fibroblast cells. **(A)** Schematic drawing of the flow experiment with a flow chamber containing NF-*κ*B oscillating cells. Through a syringe, a medium containing TNF-α is injected into the flow chamber, which initiates NF-*κ*B oscillations. **(B)** Representative example of experiment where NF-*κ*B is primarily located in the cytoplasms. **(C)** Same as B, but with NF-*κ*B is primarily located in the nuclei. **(D)** The top row images show a time-lapse of a single fibroblast cell with p65 fluorescently labeled DsRed. At temperature 32.0°C the cell is exposed to 10 ng TNF-α/ml at *t* = −2 min and a new concentration increment, to a total of 18 ng TNF-α/ml, at t = 38 min. Each image corresponds to the time points in the plot below. The graph below, shows I_*N*_/I_*C*_ ratio of the first 200 min of the data series. Data is extracted in 10 min intervals, with image examples every 30 or 40 min. **(E)** Same as D, but for temperature at 39.5°C. **(F)** Power spectrum of the I_*N*_/I_*C*_ ratio of the data presented in **(D)**. **(G)** Power spectrum of the I_*N*_/I_*C*_ ratio of the data presented in **(E)**. **(H)** Ratio (I_*N*_/I_*C*_) versus time, measured after adding 10 ng TNF-α/ml to *μ*-wells at t = −2 min. **(I)** Ratio I_*N*_/I_*C*_ versus time where 10 ng TNF-α/ml was added at t = −2 min and the concentration was again increased at t = 38 min to a total of 18 ng TNF-α/ml. **(J)** Ratio I_*N*_/I_*C*_ for the flow experiments where the TNF-α concentration is converging towards 1 ng/ml during the entire range plotted.

The flow set-up is presented schematically in Figure 1A and a more detailed illustration is shown in Figure 5 in the methods section. For these experiments, all tubing in the system would be filled up with TNF-α-free medium and the syringe connected to the inlet would thereafter inject medium with TNF-α, meaning that the TNF-α level in the flow chamber would increase. It is known from earlier experiments that the degradation of TNF-α is slow, but it is not clear whetherthe declining concentration seen both in the double, but in particular in the single-addition experiment, would affect the results. By comparing to the flow experiments, where the concentration of TNF-α was surely constant, we could control any effect of gradually decreasing concentrations of TNF-α.

In Figure 1B an example of a fluorescent image of an MEF cell at 37°C is presented at *t* = 0 min, where TNF-α was added to a concentration of 10 ng/ml at *t* = −2 min. The emitted light is from the DsRed-p65 complex and hence the image taken at *t* = 0 is a signature of the NF-*κ*B accumulation in the cytoplasm in all the cells. Additionally, we also see from Figure 1B that all nuclei are depleted from NF-*κ*B, thus showing near complete translocation of NF-*κ*B from the nucleus to the cytoplasm. This is also shown in Figure 1C, but at time *t* = 30 min. Here the cytoplasm has only a fraction of the NF-*κ*B compared to Figure 1B, and instead the NF-*κ*B has translocated into the nucleus.

To analyse the dynamics of the nuclear NF-*κ*B concentration, two different methods were used. One by doing statistics on a large number of cells (see Period-extraction method in Methods) and one for the visualization of the oscillations (see Trace-vizualisation method). With these two methods combined, we made sure that the oscillations qualitatively existed, by applying the Trace-visualization, and extracted the correct frequency of the oscillations by using the Period-extraction method. Based on the images, we were able to quantify the relative nuclear concentration of NF-*κ*B and plot this as a time series, where we at this temperature (32*°C*), observe approximately two periods in this time interval (Figure 1D, below). Here, the image series shows how the cytoplasm is bright and the nucleus is dark at *t* = 0 meaning that NF-*κ*B is accumulated in the cytoplasm (Figure 1D, above). By inspection for a higher temperature (39.5*°C*), we find approximately three periods, which can be seen both in the images and in the plot of I_*N*_/I_*C*_ ratio (Figure 1E).

We quantified the frequency of these periods, by calculating the power spectrum (see Methods), and by applying this to the time series above, we could extract frequency of 8.26 ⋅ 10^−3^min^−1^ for the time series at 32*°C*, which corresponds to a period of 121 min (Figure 1F). Similarly, we could do the same for the time series at 39.5*°C*, finding a frequency of 13.2 ⋅ 10^–3^ min^−1^ corresponding to a period of 74.1 min (Figure 1G). We further visualized the data (see Methods) of a single addition, a double addition and a flow experiment at 37°C, by showing the nuclear to cytoplasm intensity ratio, I_*N*_/I_*C*_, plotted versus time (Figures 1H–J). We note that when the TNF-α concentration is increased only once (Figure 1H), a transient peak in the NF-*κ*B concentration appears, followed by periodic oscillations with lower amplitude (Figure 1H). However, when TNF-α is added at *t* = −2 min and again at *t* = 38 min, the transient peak is followed by another peak that has higher concentration than the average amplitudes, which again is followed by oscillations with lower, and slightly decaying amplitudes (Figure 1I). Based on this, we note that when the TNF-α concentration is abruptly increased, the following NF-*κ*B peak has an larger amplitude, which is the case for both addition of one and two subsequent doses of TNF-α. These initial peaks after TNF-α addition is expected (Kellogg and Tay, 2015; Zambrano et al., 2016), but here we reveal that two successive additions will create two initial peaks with higher amplitude.

Our goal is to identify the periods of the oscillations in order to measure the difference between the three types of experiments and to finally identify how the temperature affected the period of NF-*κ*B oscillations.

We first compare the oscillations at 37.0°C, by calculating the power spectrum for each of the three types of experiments (see Methods). Here we find the periods to be.

• 105.3 min ± 21.3 min (for the single addition with n_37single_ = 12)
• 114.7 ± 29.7 min (for the double addition with n_37double_ = 10)
• 100.8 ± 10.9 min (for the flow experiments with n_37flow_ = 10)

From this, it is concluded that there is no significant difference across the three types of experiments at this physiologically relevant temperature, with Student’s t-test resulting in *p*-values > 0.05 when comparing the three populations.

**FIGURE 2:**
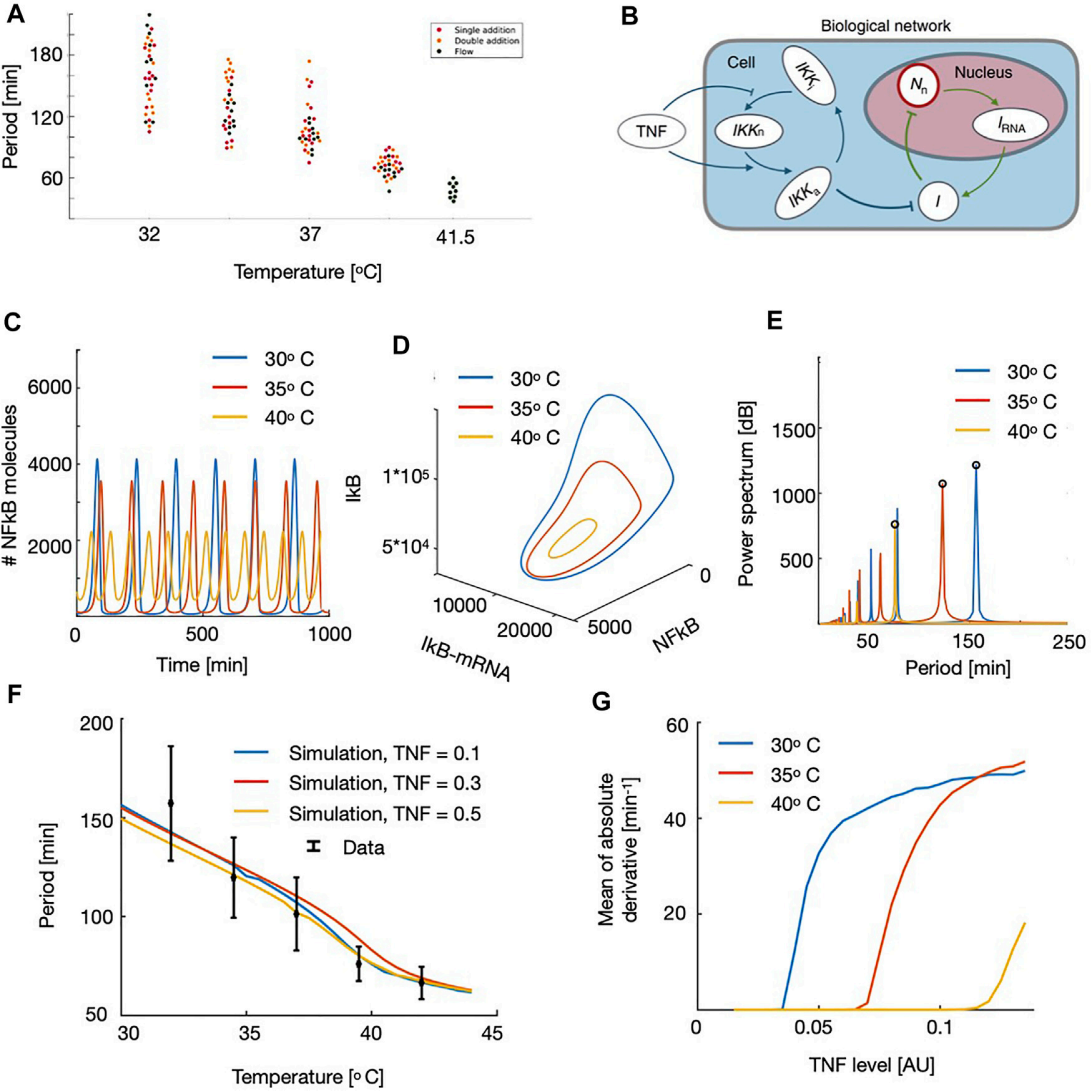
Experimental observations (A) and model data (B–F) of the temperature dependency in NF-*κ*B frequency. **(A)** Oscillation period of NF-*κ*B in fibroblast cells vs. temperature for the three different types of experiments: Single addition experiment, where 10 ng TNF-α/ml was added at t = −2 min, double addition experiment, where 10 ng TNF-α/ml was added at t = −2 min and followed by another increase of 8 ng TNF-α/ml at t = 38 min, and lastly, flow experiments, where cells were exposed to flow in a flow chamber where the TNF-α concentration was converging towards 1 ng/ml. All types of experiments were performed at 32.0°C, 34.5°C,37.0°C, and 39.5°C, and the flow experiments were in addition performed at 41.5°C (a dot corresponds to a measurement for one cell). **(B)** Schematic figure, showing the components in the NF-*κ*B network. **(C)** Time traces at three different temperatures. **(D)** Phase space for NF-*κ*B, IRNA and IkB at three different temperatures. **(E)** Power spectrum of the oscillations. The rightt most peak corresponds to the observed frequency. **(F)** NF-*κ*B oscillation period as a function of the external temperature shown for three different levels of TNF. Points in black corresponds to the experimental findings and with bars. **(G)** Absolute value of the derivative hog NF-*κ*B traces, calculated as a mean over the time series, as a function of the applied TNF level. Note that these curves rise at the onset of oscillations and thereby locates the point of the Hopf Bifurcation.

Next, we applied this method to all experiments at different temperatures. Here we measured the dynamics for temperatures down to 32°C for all three experiments. The maximum temperatures where NF-*κ*B oscillations were possible to extract, were 39.5°C for single- and double addition experiments and 41.5°C for flow experiments, indicating that the flow setup might be more robust in order to measure the dynamics during external stresses. By analysis of these time series, we find that the NF-*κ*B oscillation period decreases as a function of increasing temperature (Figure 2A). This relation was found in all three experimental conditions, emphasizing the generality if this result.

The flow experiments did result in a more smooth variation than in the experiments with single and double addition of TNF-alpha. When grouping the assays as a weighted average the periods are 160.4 ± 32.4 min and 106.8 ± 22.0 min for the 32.0 and 37.0°C, respectively. In spite of the similar results across the different types of experiments, the flow experiments resulted in more stable results, and so, for the calculation of the change of period per change of temperature, the flow experiments were used.

From these experiments, the period is constant throughout different TNF-α concentrations and throughout different methods TNF-α exposure, however, varying the temperature +4.5°C or −5.0°C compared to body temperature significantly changes the period, and is found that Δperiod/Δtemperature = −11.9 ± 2.8 min/°C.

### 2.2 Inclusion of Temperature Dependency in Mathematical Model Predicts Changes in Periods and Hopf Bifurcation

To gain insight into the mechanism behind the temperature dependency of the oscillations, we tested whether these experimental findings could be explained by the classical theory of temperature dependency of reaction rates. To describe the dynamics of the NF-*κ*B concentration, we used a mathematical model, that has been simplified in order to reduce the number of parameters, and that has previously been applied to model the dynamics of NF-*κ*B (Jensen and Krishna, 2012; Heltberg et al., 2016; Heltberg et al., 2019b; Heltberg and Jensen, 2019). In this model, we consider the NF-*κ*B inside the nucleus (*N*_*n*_), acting as a transcription factor for many proteins, including I*κ*-B. The equations take the following form:

$$\begin{array}{ll} \dot{N_{n}} & =k_{\mathrm{Nin}}\left(N_{\mathrm{tot}}-N_{n}\right)\frac{K_{I}}{K_{I}+I}-k_{\mathrm{Iin}}I\frac{N_{n}}{K_{N}+N_{n}}\\ \dot{I_{m}} & =k_{t}N_{n}^{2}-\gamma_{m}I_{\mathrm{RNA}}\\ \dot{I} & =k_{tl}I_{\mathrm{RNA}}-\alpha IKK_{a}\left(N_{\mathrm{tot}}-N_{n}\right)\frac{I}{K_{I}+I}\\ \dot{IKK_{a}} & =k_{a}\cdot TNF\cdot IKK_{n}-k_{i}IKK_{a}\\ \dot{IKK_{i}} & =k_{i}IKK_{a}-k_{p}IKK_{i}\frac{k_{\mathrm{A20}}}{k_{\mathrm{A20}}+\left[A20\right]\cdot TNF}\\ & IKK_{n}={\left[IKK\right]}_{\mathrm{tot}}-IKK_{a}-IKK_{i} \end{array}$$

Here, *N*_*n*_ is the nuclear NF-*κ*B concentration, *I*_*m*_ is the IkB mRNA level, and *I* is the concentration of cytoplasmic I-*κ*B protein. I*κ*-B kinase (IKK) is activated by the external signal TNF and causes eventual targeted degradation of I*κ*-B when it is bound to NF-*κ*B. IKK has the forms: neutral (n), active (a) and in-active (i). The network is schematized in Figures 2B, and a more detailed description can be found in the methos section, where all the parameters are listed as well (note that in the model section we use the notation TNF instead of TNF-α for simplicity).

We now include the temperature dependency into the simulations. In the model, we have 9 rates, and these were all made temperature dependent (See Table 1 in Methods). We assumed that the fastest ones: *α*, *k*_*Nin*_ and *k*_*t*_ followed a Smoluchowski dependency (i.e., they are diffusion limited, *k* ^+^ ∝ *D*(*T*)), whereas the others followed an Arrhenius dependency (i.e., they are reaction limited $k^{+}\propto e^{\frac{E}{k_{B}T}}$ ). This distinction is due to the fact that all particles need to find their targets, and in that sense they are all subject to the timescale of diffusive first-passage times. If it additionally takes a very long time to complete the chemical binding/reaction, there is another timescale included which follow the Arrhenius equation and if this is large, one can neglect the effect of the diffusion timescale. In this way, if we assume molecules diffuse approximately at same rate, the slow binding constants will be governed by the Arrhenius equation. It should be noted that the results do not differ significantly, if the Smoluchowski dependent rates are all turned into Arrhenius rates. It should be noted, that while the activation energy (E) in the Arrhenius equation For details see Methods.

**TABLE 1:** Default values of parameters in the model. Here we assume that the slowest rates are governed by the Arrhenius equation, whereas the fastest rates will follow the temperature dependency of the Smoluchowski rate (Diffusion limited). The temperature dependencies and their activation energies are listed when applicable.

Parameter in paper	Default value	Temperature dependency	$\ln\left(\frac{1}{k_{0}^{+}}\right)\frac{1}{A}$
*k*_*Nin*_	5.4 min^−1^	Diffusion limited	NA
*k*_*Iin*_	0.018 min^−1^	Reaction limited	20
*k*_*t*_	1.03 (*μ*M)^−1^.min^−1^	Diffusion limited	NA
*k*_*tl*_	0.24 min^−1^	Reaction limited	1
*K*_*I*_	0.035 *μ*M	NA	NA
*K*_*N*_	0.029 *μ*M	NA	NA
*γ*_*m*_	0.017 min^−1^	Reaction limited	1
*α*	1.05 (*μ*M)^−1^.min^−1^	Diffusion limited	NA
*N*_*tot*_	1 *μ*M	NA	NA
*k*_*a*_	0.24 min^−1^	Reaction limited	1
*k*_*i*_	0.18 min^−1^	Reaction limited	20
*k*_*p*_	0.036 min^−1^	Reaction limited	20
*k*_*A20*_	0.0018 *μ*M	NA	NA
[*IKK*]_*tot*_	2.0 *μ*M	NA	NA
[*A*20]	0.0026 *μ*M	NA	NA

With this set-up we were ready to simulate the dynamics of NF-*κ*B at different temperatures. By varying the temperature we found that the NF-*κ*B oscillations were highly affected by the change in the temperature level and that low temperatures led to large periods and amplitudes (Figure 2C). It should be noted that it is possible to change the period of oscillations, without affecting the amplitudes by simply rescaling the time dependent parameters. However, since the temperature dependency acts both on the diffusion limited and the reaction limited parameters, we do not obtain this scaling and as a result the amplitudes are affected by this temperature variation. We visualized this further in the three-dimensional phase space spanned by the NF-*κ*B, IRNA and IkB. Here we note that the change in temperature affects the entire size of the limit cycle and for high temperatures the limit cycle shrinks, leading to faster oscillations (Figure 2D). To quantify these oscillations, we calculated the power spectrum, by applying the FFT algorithm, finding the leading frequency of the time series. We note that multiples of this oscillation appear as well, but taking the maximal value of the power spectrum we find the correct frequency of the oscillations (Figure 2E). We now used this algorithm to calculate the period as a function of the applied temperature. Here we found that the curve decreases monotonically for increasing temperature, which is what we would expect by observing the time series, and by comparing these results to the experimental observations we find a striking compliance (Figure 2F). We tested this for different values of TNF baseline levels, and here we found similar patterns indicating that this result is quite robust and not sensitive to our initial choice of external TNF level (Figure 2F). We also conclude that this scaling with temperature is not dependent on the followed protocol, which solidifies the robustness of this study and can be interpreted as TNF-α is extremely slowly degraded in these environments. Since the temperature affected the period of the oscillations, we were also interested if this meant that changes in the temperature could also induce oscillations and thereby affect the point of the onset of oscillations (i.e. the Hopf bifurcation). Here we found to our surprise that decreasing the temperature, would lead to oscillations for smaller values of the external TNF levels (Figure 2G). This means that it would be possible to use regulations of temperature to induce or stop the oscillations in living organisms.

### 2.3 Control of Downstream Protein Production by Changing the Temperature

**FIGURE 3:**
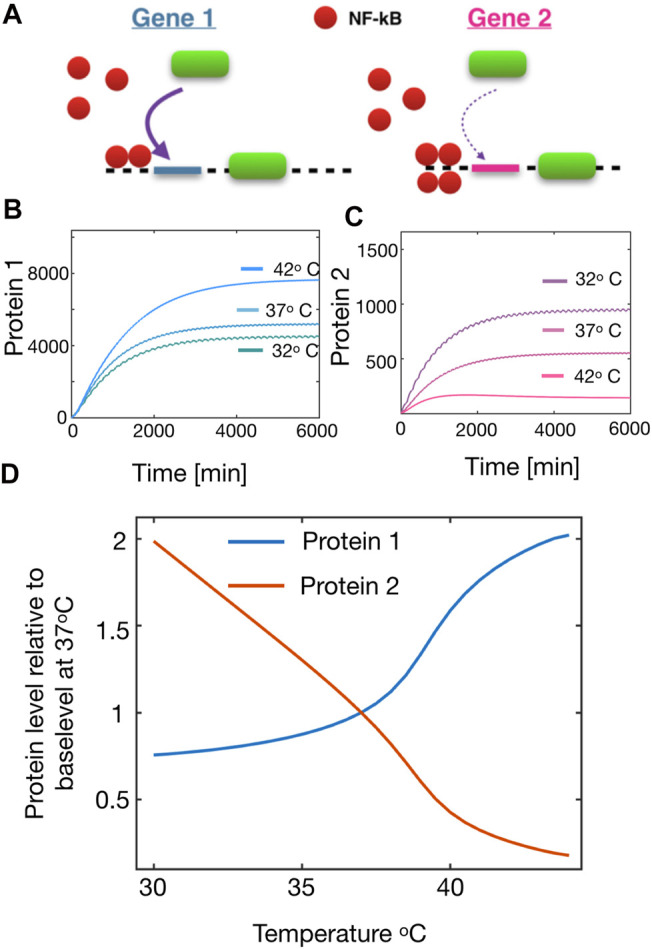
Temperature dependency of the protein production in downstream genes from *NF* − *κB* oscillations. **(A)** Schematic figure showing the stimulation of a high affinity gene (left) and a low affinity gene (right). **(B)** Protein production of Protein 1 (from a high affinity gene) at three different temperatures. **(C)** Same as **(B)** but for Protein 2 (from a low affinity gene). **(D)** Relative steady state production of Protein 1 and Protein 2 respectively as a function of temperature.

As we have established how the dynamics of NF-*κ*B could vary with different temperature levels, we wanted to investigate how this could affect the downstream production of proteins stimulated by NF-*κ*B. Here we used a previously suggested model (Heltberg et al., 2016), where all genes are divided into groups based on their affinity and cooperativity from stimulation with NF-*κ*B. We assume that NF-*κ*B can bind to an enhancer or operator region, and can form complexes to bind the RNA polymerase, with different affinity, depending on the gene (schematically shown in Figure 3A). We describe the transcription and translation of each gene, labelled *i* = 1, 2, 3, … , using the differential equations [see (Heltberg et al., 2019b)]:

$$\begin{array}{ll} {\dot{m}}_{i}= & \gamma_{i}\frac{N^{h_{i}}}{N^{h_{i}}+K_{i}^{h_{i}}}-\delta_{i}m_{i},\\ {\dot{P}}_{i}= & \Gamma_{i}m_{i}-\Delta_{i}P_{i}. \end{array}$$

Here, the *m*_*i*_ represent the mRNA level transcribed from gene *i*, and *P*_*i*_ represents the concentration of proteins produced from the corresponding mRNA. The first term in the equation for the mRNA is known as a Hill function; the canonical way to describe the protein production for genes governed by transcription factors where each gene has a specific Hill coefficient and effective affinity (Werner et al., 2007; Kaplan et al., 2008; Maienschein-Cline et al., 2010; Mengel et al., 2010; Sneppen et al., 2010).

The effective affinity *K*_*i*_ is a parameter that combines the strength of binding of the transcription factor to the operator/enhancer region, the strength of binding of RNA polymerase to the promoter and transcription factor, as well as the effect of DNA looping that may be needed to bring the enhancer/operator close to the promoter region. Operationally, *K*_*i*_ sets the concentration of NF-*κ*B that results in 50% of maximal gene expression enhancement.

With this set-up, we simulated the model of NF-*κ*B with varying the temperature as shown in Figure 2, and measuring the produced proteins. For simplicity we will only consider two proteins, Protein 1 and Protein 2, being stimulated from a High and Low affinity gene respectively. From the model we see that as we increase the temperature, the steady state level of Protein 1 is enhanced (Figure 3B). However as we assessed the steady state level of Protein 2, we realised that this was significantly reduced and thus these types of proteins would be up-regulated if the cell could lower the temperature (Figure 3C). The reason for this change in the protein level, is predominantly due to the variation in the amplitudes of NF-*κ*B. The low affinity genes (here Protein 2) will rarely be expressed, unless very high levels of transcription factor available, which happens during the large transient pulses. However, due to the spiky oscillations in this regime, NF-*κ*B remains very low for long periods of time which will cause a decrease in the high affinity genes that are otherwise always expressed. In this way the tuning of oscillations might alter the overall protein production. With this information we simulated the steady state protein level of both proteins as a function of temperature, and here we found a very interesting crossover effect (Figure 3D) which indicates that proteins from High affinity can be up-regulated as one increase the temperature whereas proteins from Low affinity genes are monotonically decreased for increasing temperature. This result highlights the fascinating prospect, that one can use temperature as a regulator for the downstream production of proteins.

### 2.4 Synchronization and Chaotic Dynamics Emerges From Temperature Oscillations

At this point, we had established that temperature variations affect the oscillatory properties of NF-*κ*B. Therefore, we hypothesized that a periodically varying temperature could lead to highly complex dynamics in the nuclear NF-*κ*B. Mathematically, the introduction of periodically varying temperature variations turns the system into two coupled oscillators (Jensen et al., 1983; Jensen et al., 1984; Heltberg et al., 2016; Heltberg et al., 2021), where NF-*κ*B is an internal oscillator, stimulated by an external temperature oscillator. In the experiments, we found that cells would survive and remain oscillatory for temperature variations of ± 5*°C*, and in order to remain close to the experimental observations, we allow the amplitude of the temperature oscillations to be maximally 5*°C*.

**FIGURE 4:**
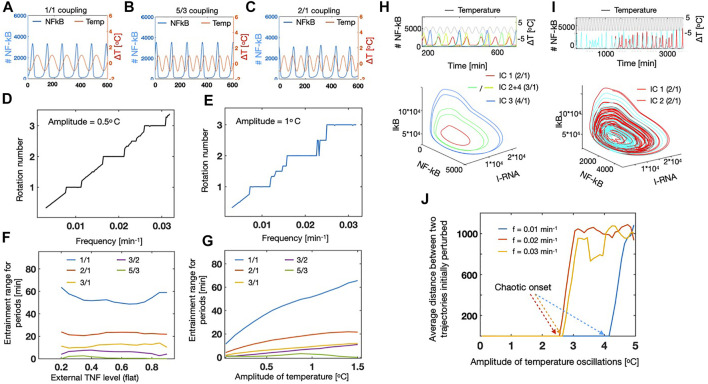
Temperature oscillations lead to entrainment and chaotic dynamics for NF-*κ*B. **(A)** Oscillations in NF-*κ*B (blue, left axis) coupled to temperature (red, right axis). Temperature amplitude = 1*°C* and frequency (*f*_*tmp*_) = 0.01[ min^−1^]. **(B)** Same as A, but with temperature frequency = 0.018[ min^−1^] resulting in a 5/3 coupling. **(C)** Same as A, but with temperature frequency = 0.02[ min^−1^] resulting in a 2/1 coupling. **(D)** Rotation number measured as *f*_*tmp*_/*f*_*NF*−*κB*_ as a function of the oscillation frequency for the temperature. Amplitude of temperature = 0.5*°C*. **(E)** Same as D, but with amplitude of temperature = 1*°C*. **(F)** Width of the dominating steps in the figures D + E as a function of the external TNF level. Amplitude of the temperature oscillations = 1*°C*. **(G)** Width of the dominating steps in the figures D + E as a function of the amplitude of temperature oscillations. TNF level = 0.2. **(H)** Dynamics of NF-*κ*B for different initial conditions (indicated by colours) as a function of time (above) and in the phase space spanned as *I*_*RNA*_ *κB* and I*κ*B **(I)** Same as H, but for two initial conditions separated by 10^–4^%. **(J)** Mean distance of the trajectories, initially separated by 10^–4^% as a function of the amplitude of the temperature oscillations. Curves shown for three different frequencies of temperature oscillations.

To a start, we oscillated the temperature with an amplitude of 1*°C* and by varying the frequency we observed that different entrainment modes emerged. First we found the 1/1 coupling, which means that one full period of the temperature corresponds to one period of the NF-*κ*B system, where the phases are locked, Figure 4A [see e.g., (Heltberg et al., 2016; Heltberg et al., 2021)]. By varying the period, it became clear that this temperature dependency could lead to entrainment for different rational numbers, for instance with a 5/3 coupling (Figure 4B) and strong 2/1 coupling (Figure 4C). A 5/3 coupling corresponding to a synchronized situation where the NF-*κ*B signal performs 3 cycles while the temperature performs 5 cycles (see Figure 4B). Similarly, the 2/1 coupling corresponds to a state with one NF-*κ*B cycle for each two temperature cycles (Figure 4C). Based on these first observations, we varied the frequency and measured the rotation number of the system. This rotation number we define as the (externally fixed) frequency of the temperature divided by the (measured) NF-*κ*B frequency. By doing this we were able to extract the resulting plot known as a Devil’s staircase (Figures 4D,E) for two different values of the external amplitude. Here we find entrainment plateaus (horizontal regions), were the NF-*κ*B frequency is entirely determined by the temperature frequency. This means that inside these regions, one can completely control of behaviour of the NF-*κ*B oscillations and either speed up or slow down the dynamics. We note by comparing Figures 4D,E that the dominating plateaus grow in range, as we increase the amplitude from 0.5*°*C to 1*°C*. We therefore wanted to measure the width of these entrainment regions especially for the dominating ones. First we tested how this was affected by the external level of TNF, and we found that even though small variations occurred, these entrainment regions were quite stable and robust to changes in the TNF level (Figure 4F). This is a promising observation, since it allows future experiments *in vivo* and *in vitro* to focus on the changes in temperature, without worrying about the small differences in the levels of TNF. Next we increased the temperature amplitudes, and here we found that all the dominating regions were growing, whereas the smaller rational regions (such as 5/3) loose their stability as they are being “squeezed” out by the dominating ones (Figure 4G).

It has previously been observed that for large amplitudes of oscillatory TNF, the dynamics of NF-*κ*B, could show “modehopping” which corresponds to transitions between two stable limit cycles (Heltberg et al., 2016; Heltberg and Jensen, 2019). We were interested if this was also the case, if we applied temperature oscillations, and we realised that already for amplitudes of 2*°C*, multistability occured, and we were even able to find three stable limit cycles by varying the initial conditions of the simulation (Figure 4H). We note that for some of these limit cycles, the dynamics could entrain in different phases even though they were part of the same attractor in the phase space (see IC2+IC4 in the time series above in Figure 4H). This observation also indicated that the applied amplitude was above the “critical value”, and due to a theorem of Poincare, above this line various complex dynamics could emerge and in particular chaotic dynamics (Jensen et al., 1984). Therefore, we investigated this by increasing the external amplitude, and found that chaotic dynamics could emerge for amplitudes around 2.5*°C*. We visualized the chaotic dynamics, by simulating the system with identical parameter values, and initial conditions only separated by 10^–4^%. By studying the time series, it is clear that even though they show completely similar dynamics for a long time, then the time series evolve completely differently (Figure 4I above). We also visualized this in the three-dimensional phase space, and could see that the trajectories moved on a strange attractor (Figure 4I below). Finally we were interested in studying for which values of the temperature oscillations, one expect to see the chaotic transition. To quantify this, we measured the average distance between two trajectories only separated by 10^–4^% in the initial conditions. Using this measure we observed that for relatively large frequencies of the temperature oscillations (periods of 30–50 min), we found a chaotic transition for $\approx 2.5^{o}$ C, whereas for temperature oscillations with a period of 100 min, we would expect the amplitude to be around $\approx 4.1^{o}$ C before the chaotic transition emerges (Figure 4J). However we note that all of these values are in agreement with the observed temperature variances for the flow experiment and we therefore predict that one could induce chaotic dynamics by applying this experimental set-up described above.

## 3 Discussion

Temperature is known to vary in living organisms as a response to external stresses, but a mechanistic understanding of how this can affect the protein production and control transcription factor dynamics has been scarcely investigated. In this work, we have shown how the transcription factor NF-*κ*B can change it is properties of oscillations by varying the temperature in a realistic range where the cells can still survive and maintain oscillations. We use theories of statistical physics and in particular the Smoluchowski equation to predict how the individual rates are affected by a changing temperature, and show that these are sufficient to describe the results of the experimental data. We further use this calibrated model, to predict how different families of downstream genes are affected by a change in temperature and we point out a crossover effect with strong regulatory implications. Finally, we show that temperature can be used as a strong source to induce more complex dynamics to the NF-*κ*B and we suggest that this can be used as a key measure to investigate the role of dynamics in transcription factors on the downstream gene production.

The role of temperature dependency on the NF-*κ*B oscillations has previously been studied (Harper et al., 2018), where an increasing frequency for increasing temperature was also observed, even though this effect was only investigated for a smaller temperature range than investigated here. One of the findings in (Harper et al., 2018), was an A20 dependent mechanism that was a key element in the perturbation of NF-*κ*B frequency. Here, we apply a model where A20 is assumed to be constant, which is originally based on the findings of Ashall et al. (2009), in which they argue that there is a range of constitutive A20 expression values that can functionally replace A20 negative feedback. Therefore, the mechanism of the temperature dependency may not be linked directly to the dynamics of A20, however, two of the most impactful parameters (*k*_*i*_ and *k*_*p*_, see Table 1) both affect the part of the network that A20 also acts on. Therefore, our results are generally in agreeing with those of Harper et al., however, they suggest that on a fundamental level, it is the effective turnover of IKK that mediates the temperature dependency in the oscillations of NF-*κ*B.

Our findings reveal a fundamental temperature dependency in the oscillations of NF-*κ*B and it is therefore intriguing to speculate what the functional role of this might be. We hypothesize that the alterations in the oscillations might affect and stimulate different groups of genes. However, the most studied oscillator in biological systems, the circadian clock, is known to be robust to changes in temperature (Leloup and Goldbeter, 1997; Hong et al., 2007), and therefore it could be argued that it would be beneficial to biological oscillators to adopt this robustness. We believe that a key difference between these biological oscillators is that the NF-*κ*B oscillations should be regarded as a response and therefore it would be advantageous to be able to tune this response to different stimuli and the temperature in particular. On the other hand, the circadian oscillator is basically a very advanced clockwork, where robustness is the key to maintain time keeping. Finally, in the tissues where the NF-*κ*B oscillator is important, the temperature is well-regulated and thereby changes in temperature would be a parameter to trigger a response where the NF-*κ*B oscillator would be part of it.

The finding that oscillations emerge by lowering the temperature while keeping TNF at a constant low level, combined with the fact that the low affinity genes are in general enhanced in the low-temperature regime, suggests a potential functional role of the temperature stimulation on NF-*κ*B. Since NF-*κ*B stimulates several hundreds of genes downstream, it is potentially important to stimulate a subset of genes, while lowering other families of genes. The mechanism suggested here can create this type of cross-over effect by simply changing the temperature for the cells. In this way an entire cascade can potentially be initiated by lowering the temperature, which will lead to an up-regulation of specific groups of genes that are otherwise not expressed.

Even though chaotic dynamics has been studied theoretically and experimentally for more than 50 years, it has still not been shown to exist and play a role in cellular dynamics. Complex phenomena such as synchronization has been shown to exist (Danino et al., 2010; Kellogg and Tay, 2015; Heltberg et al., 2016), and even modehopping that reveals the existence of multistable cycles, when the amplitude of the external oscillator has been sufficiently increased. Theoretically this should also guide a way to induce chaotic dynamics (Jensen et al., 1983; Jensen et al., 1984; Heltberg and Jensen, 2019), however often cells have trouble surviving the transiently high concentrations of TNF-α, and therefore it has been difficult to predict how chaotic dynamics might be investigated for such systems. Our results predict that temperature oscillations is a simple and effective way to induce highly complex dynamics when the temperature is oscillated externally. Since our experimental results reveal that cells can survive and maintain oscillations under temperature variations of $\approx \pm 5^{o}C$ , this system should be stable enough to induce chaotic dynamics under oscillations with temperature amplitudes in this range. If future experiments succeed in using the temperature oscillations, it is possible to distinguish chaos from oscillatory behaviour with stochastic noise; see for example (Gilmore and Lefranc, 2002; Amon and Lefranc, 2004; Heltberg et al., 2021). A potential functional role of chaos is that very large amplitudes will emerge which might be enough to further stimulate the low affinity genes even further. This is studied in details in (Heltberg et al., 2019b), and the chaotic dynamics revealed by the temperature stimulation should lead to similar enhancement of not only low affinity genes by also of protein complexes with subunits from both low- and high affinity genes. We surmise that future directions of experimental investigations might focus on developing experimental protocols to study the emergence of complex dynamics in cells and how this affects and regulates the cellular machinery under different external stresses.

Temperature presents a fundamental, physical property with the potential to control and regulate the dynamical properties of protein concentration in cells. It is our hope that this work will inspire theoretical and experimental explorations these prospects of transcription factors, as the system is affected by either constant or dynamically varying temperatures in living cells.

## 4 Methods

### 4.1 Cell Culturing

All cells used in this article were stably transduced 3T3 mouse fibroblast cells obtained from Tay et al. (2010). Using lentivirus, DsRed was introduced into the genome of the cells, resulting in the expression of the fusion protein DsRed-p65. This allowed for tracking of p65 when shuttling between cytoplasm and nucleus of the cells. These cells also had the nuclear marker H2B-GFP which was not utilized in the presented data of this article.

Cells were incubated in 5.0% CO_2_ at 37.0°C in a HERA CELL VIOS 160i CO_2_ incubator. For culturing Gibco™ DMEM culture medium with high glucose, L-Glutamine, phenol red, and no Sodium Pyruvate and no HEPES were used. While performing experiments this medium was substituted with Gibco™ DMEM culture medium with high glucose, HEPES, L-Glutamine, no phenol red, and no Sodium Pyruvate. To both media +10% Fetal bovine serum (FBS) and +1% Penicillin-Streptomycin (PS) was added.

### 4.2 Single Well Experiments

48 h prior to the experiments fibroblast cells were seeded on collagen-coated, *γ*-radiated, 35 mm, No. 1.5 glass bottom microwell dishes (Mat Tek, 2021). The microwells were brought from the 37.0°C, 5% CO_2_, high humidity incubator into the incubation chamber of the microscope with an atmosphere of 5% CO_2_, high humidity, and a specific target temperature. Approximately 15 min after, when the temperature had stabilized, the 10 ng TNF-α/ml was added to a total of 40 ng TNF-α. One to two minutes thereafter the fluorescence microscope time-lapse was initiated. This initiation was defined as time = 0 min in the experiments. Timelapse images were obtained every 10 min where 10–12 positions were captured at each time point throughout the entire timelapse series. In the experiments where TNF-α was added twice, the first addition was once again 10 ng TNF-α/ml to a total of 40 ng TNF-α, and the second addition took place one to two minutes before the fourth set of timelapse images at t = ≈ 38 min, and consist of a total increase of 8 ng TNF-α/ml. At the second addition 50 ng TNF-α was added and the total TNF-α added to these experiments were 90 ng TNF-α.

### 4.3 Flow Experiments

**FIGURE 5:**
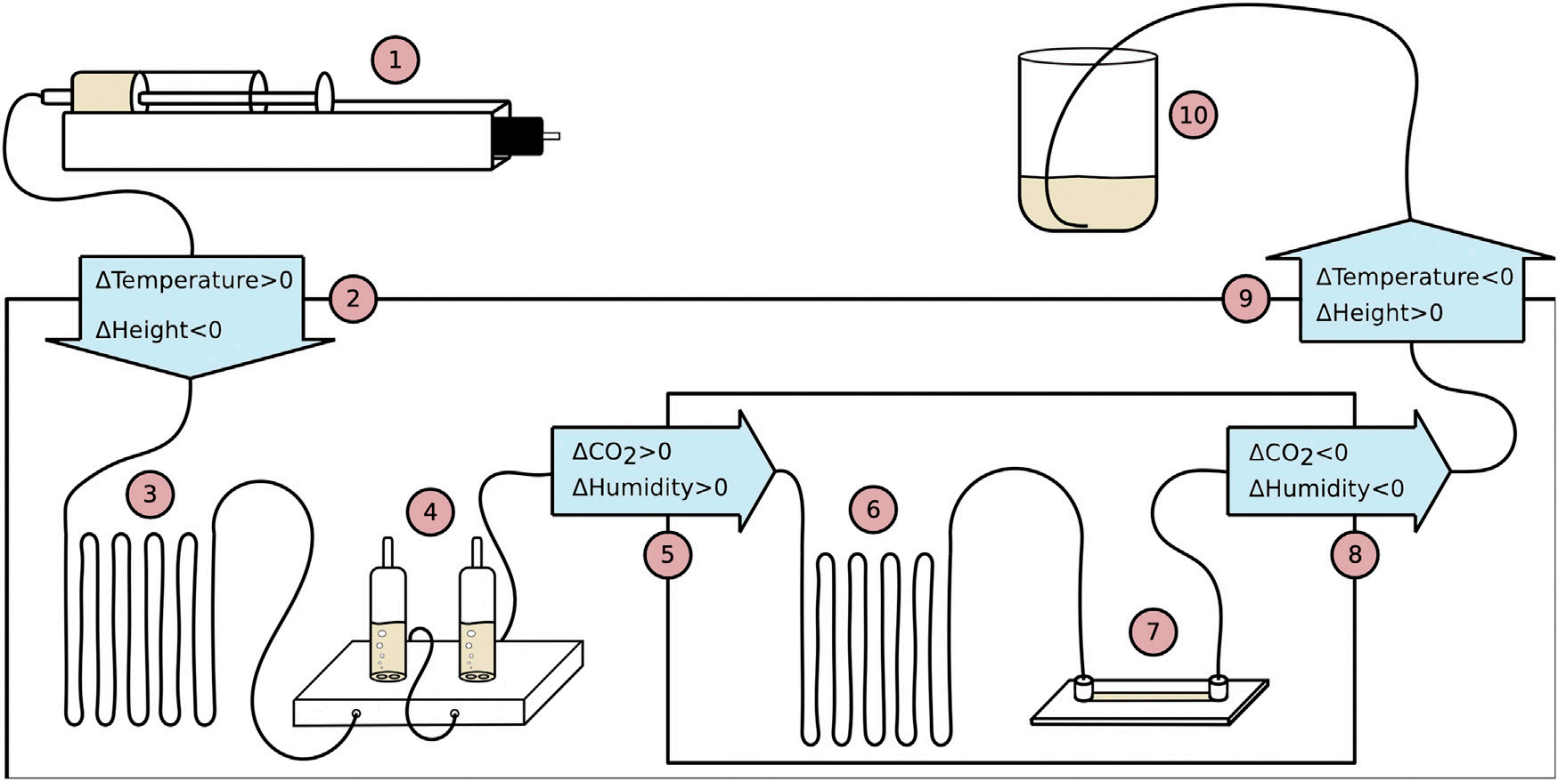
Schematic drawing of the flow system with numbers added, indicating how the medium flows through the system, chronologically. The solid line connecting the devices is a gas permeable silicone tubing. 1) Pump injecting medium into the system at a rate controlled by a computer *via* Labview software. 2) Medium flows into the microscope enclosure. Medium changes from room temperature to target temperature −2.0°C and changes from the starting height to the same level as all devices inside the microscope enclosure (ΔHeight ∼ −40 cm). 3) 1.0 m of extra tubing lets the medium reach target temperature −2.0°C before entering the bubble trap. 4) Two bubble traps capturing excess gas in the tubing. 5) Medium enters incubation chamber and the atmosphere changes from room atmospheric CO_2_ and room atmospheric humidity to 5% CO_2_ and high humidity while also the temperature increases with 2.0°C to the target temperature. 6) Extra tubing allows the medium to adjust to CO_2_ and temperature. 7) Perfusion chamber where the fibroblast cells grow and the microscopy data is collected. 8) Medium exits incubation chamber and CO_2_ and humidity changes to atmospheric levels. Temperature changes to target temperature −2.0°C. 9) Medium exits microscope enclosure and temperature changes from target temperature −2.0°C to room temperature. Height of medium is increased (ΔHeight ∼ +30 cm). 10) Medium flows out of the flow system and into a glass beaker.

When performing flow experiments illustrated in Figure 5, a major task is to prevent gas formation in the perfusion chamber where cells grow. The media utilized in the experiments have to be stored at room temperature but the temperature of the perfusion chamber is approximately 10–20°C higher than room temperature, and since Gas is extruded from a liquid when it is heated up, gas formation in the perfusion chamber is an inherent problem to flow chamber (MBR) flow experiments. If the perfusion chamber is filled up with gas, even temporarily, in the order of minutes, cells will be injured. They will not be able to fully recover and likely cells will undergo necrosis. If cells survive, however, our pilot experiments showed that nuclear translocation of NF-*κ*B shuttling was prevented. Following, the extensive measures required to prevent gas formation are described.

Prior to the initialization of the experiments, the medium was degassed for 90 min. Subsequently, the medium was brought to a flow hood and filtered using a 0.2 *μ*m filter to remove biological contaminants from the degassing process. The medium was separated into two portions. One where TNF-α was added and one that was left for filling up the flow system before starting the experiment. From degassing, the medium was cooled down, so before injecting it into the flow system5, consisting of tubing, bubble traps, and perfusion chamber, the medium was heated up to room temperature in a sealed falcon tube to prevent new gas from being dissolved in the medium while the medium was heating up. The entire flow system was then filled up with medium in steps and assembled inside the flow hood. In each bubble trap, 3 ml of medium was injected, leaving 2 ml in each bubble trap for air. The entire flow system was then brought to the microscope set-up and inserted into the microscope enclosure. The chamber and 1 m of tubing were inserted into the incubation chamber, an inlet was connected to the controllable pump outside the enclosure and an outlet was inserted into a glass beaker.

The inlet and outlet of the system were elevated compared to the other devices in the system. This was to create increased pressure in the perfusion chamber and tubing situated in the incubation chamber in order to minimize gas formation. In between the injection and the perfusion chamber, the temperature increases in two steps. The first increment is close incubation chamber, where the temperature is reached, which serves two purposes: 1) It stabilizes the temperature surrounding the incubation chamber, which will minimize temperature fluctuations. 2) When gas forms in the medium due to increased temperature, the temperature has already increased significantly before entering the bubble trap and most gas will be trapped herein. In between the microscope enclosure and the bubble trap, extra tubing is inserted to make sure the temperature of the medium is increased and to give the medium time to extrude gas before entering the bubble trap. After the bubble trap, the medium flows into the incubation chamber, where the medium and perfusion chamber is exposed to the target temperature, 5% CO_2_, and high humidity.

### 4.4 Microscopy

A Nikon inverted fluorescence microscope was utilized throughout these experiments (Nikon, 2021). A Lumencor Sola Light Engine (Lumencor, 2021a) solid-state illumination provides fluorescent light combined with excitation- and emission filters in the microscope at 575 and 641 nm, respectively. Bright-field images are provided by the build-in lamp in the microscope. Images are captured with an Andor Neo sCMOS model DC-152Q-COO-FI camera (Lumencor, 2021b).

### 4.5 Temperature Control

Temperature control inside the microscope enclosure was provided by Oko lab incubation unit model H201-T-0016 (Oko Cage Incubator, 2021). The temperature of the incubation chamber, provided by the Warner Duel Automatic Temperature Controller TC-344B (Warner, 2021), was 2.0°C higher than the temperature in the microscope enclosure. Each of its two controllers had two heaters, a control thermometer, and a monitor thermometer. The two heaters connected to one of the controllers were mounted to the bottom stage of the outsides of the incubation chamber and two heaters connected to the other controller were mounted to the inside of the incubation chamber. The heaters outside the incubation chamber were constantly exposed to 6 V from the controller, while the heaters inside were continuously controlled by a loop, meaning the temperature inside the incubation chamber could be set to a constant value. The control thermometer of the inside heaters was taped on top of the flow slide in the case of flow experiments and taped onto the bottom of the inside of the incubation chamber in the case of micro dish experiments. During flow experiments, the temperature of the cells inside the perfusion chamber was assumed to be equal to the temperature on top of the flow slide, where the control thermometer was attached. In micro dish calibration experiment it was shown that the temperature inside a micro dish containing 4 ml of water was 1.0°C lower than a thermometer attached to the bottom of the inside of the incubation chamber, so in these experiments, the temperature of the Warner temperature controller was set to 1.0°C higher than the target temperature.

### 4.6 Data Analysis

A semi-automatic Matlab program was built for the analysis, where nuclear light intensity (I_*N*_) and cytoplasm intensity (I_*C*_) were measured and an oscillation period was extracted for a single cell at the time, by taking the ratio of I_*N*_/I_*C*_. Prior to being analysed in the Matlab program, cells with clear oscillation patterns were chosen from the visual image analysis in Fiji. In the Matlab program, the average light intensity of a circular area for each of the nucleus and cytoplasm was selected for each time frame, corresponding to a change in time of 10 min. Depending on the size of the cell, the measured area would be between 3.1 and 8.7 *μ*m^2^, corresponding to a circle with a diameter between 2 *μ* m and 3.3 *μ*m, and corresponding to between 27 pixels and 79 pixels for each measured nuclear or cytoplasm intensity.

This approach was used throughout all data analysis, but it was found to be an advantage to both have a Trace-vizualisation method and the Period-extraction method for the total data analysis. The Trace-vizualisation method would visualize the oscillations to give an understanding of the shape of the oscillations as well as give an understanding of how well the system behaved. This method was very time-consuming, so another method, namely the and Period-extraction method was used to extract statistics on oscillations of a high number of cells in a more time-efficient manner.

### 4.7 Trace-Vizualisation Method

With the analysis in Fiji described above, a single area from the cytoplasm and a single area from the nucleus were extracted. Each cell was analysed in a time span of between 90 and 770 min, equivalent to be between 2 and 11 oscillations.

The Matlab program would then do a power spectral analysis of I_*N*_/I_*C*_—mean(I_*N*_/I_*C*_) and extract the frequency value of the highest peak, see Figures 1D–G). The inverse of this frequency value would then correspond to the most significant period in the data series. However, in some cases, a peak that did not correspond to the cell’s oscillations was the highest peak. In these cases, the high peak near the visually observed oscillation was chosen as the frequency for extracting a period.

### 4.8 Period-Extraction Method

In this analysis, four areas of the same size as described above were chosen for both the cytoplasm and for the nucleus. This made the total areas for the calculations of the I_*N*_/I_*C*_ ratio larger resulting in local intensity variations being minimized. The I_*N*_/I_*C*_ ratio was then smoothed with the Matlab Smooth Function, with a span = 4. From experimental errors, the I_*N*_/I_*C*_ data had intensity variations larger than the typical oscillation period. These variations were filtered out by taking the Matlab Smooth Function with span = 13. The Matlab Smooth function with span = 13 was then subtracted to the Matlab Smooth Function with span = 4, which resulted in a data series well suited for qualitative understanding of the oscillations.

### 4.9 Temperature Dependency

With the mathematical input based on Smoluchowski equations (see Supplementary Materials) we define how we expect the rates should change according to a change in temperature. As standard we set $\beta =\frac{1}{k_{B}T}$ and start out by considering the limit of low absorbing rate (i.e., *κ* ≈ 0), where we denote *U*(*R*_0_) = *E* as the activation energy and $4\pi R_{0}^{2}\kappa =A$ as an activation constant. With this we obtain the on rate by:

$$k_{\kappa \approx 0}^{+}=Ae^{-\beta E}$$ (1)

Now differentiating with respect to temperature gives:

$$\frac{\partial k^{+}}{\partial T}=Ae^{-\beta E}\frac{E}{k_{B}T^{2}}=k^{+}ln\left(\frac{1}{k^{+}}\right)\frac{1}{A}\frac{1}{T}$$ (2)

Assuming linearity around the value of T, we can rewrite the differential equation into a difference equation:

$$\frac{k_{n}^{+}-k_{0}^{+}}{T_{n}-T_{0}}=k_{0}^{+}ln\left(\frac{1}{k_{0}^{+}}\right)\frac{1}{A}\frac{1}{T_{0}}\;\;\Leftrightarrow$$ (3)

$$k_{n}^{+}=k_{0}^{+}\left(1+ln\left(\frac{1}{k_{0}^{+}}\right)\frac{1}{A}\frac{\Delta T}{T_{0}}\right)$$ (4)

The constant A, is known as the exponential prefactor in the Arrhenius equation, and this is a free parameter that we do not have knowledge about. In the equations, we obtain the scaling factor $\ln\left(\frac{1}{k_{0}^{+}}\right)\frac{1}{A}$ and these are shown in the Table 1. Of the 6 parameters that follow the Arrhenius equation, 3 are kept to unity, whereas other have a larger impact and these are set to 20. In order to reduce the number of varying parameters, we have fixed this value for all three impactful parameters, but we note that this is arbitrarily chosen and we would obtain similar results if this value were smaller or larger.

Next consider the case of diffusion limited reactions. This is the other limit in the equations above, where we also assume there is no potential. Here we obtain the maximal rate as:

$$k^{+}=4\pi D_{0}R_{0}$$ (5)

If we apply the Einstein-Stokes relation we find the temperature dependency in *D*_0_

$$D_{0}=\frac{k_{B}}{6\pi \eta r}T$$ (6)

This means that the rate would simply follow the temperature according to:

$$\frac{k_{n}^{+}-k_{0}^{+}}{T_{n}-T_{0}}=4\pi R_{0}\frac{k_{B}}{6\pi \eta r}\;\;\Leftrightarrow$$ (7)

$$k_{n}^{+}=k_{0}^{+}\left(1+\frac{\Delta T}{T}\right)$$ (8)

### 4.10 The NF-κB System

In this model, we consider the NF-*κ*B inside the nucleus (*N*_*n*_), acting as a transcription factor for many proteins, including I-*κ*B. The equations are repeated here with the addition of an oscillating TNF value:

$$\dot{N_{n}}=k_{\mathrm{Nin}}\left(N_{\mathrm{tot}}-N_{n}\right)\frac{K_{I}}{K_{I}+I}-k_{\mathrm{Iin}}I\frac{N_{n}}{K_{N}+N_{n}}$$ (9)

$$\dot{I_{\mathrm{RNA}}}=k_{t}N_{n}^{2}-\gamma_{m}I_{\mathrm{RNA}}$$ (10)

$$\dot{I}=k_{tl}I_{\mathrm{RNA}}-\alpha IKK_{a}\left(N_{\mathrm{tot}}-N_{n}\right)\frac{I}{K_{I}+I}$$ (11)

$$\dot{IKK_{a}}=k_{a}\cdot TNF\cdot IKK_{n}-k_{i}IKK_{a}$$ (12)

$$\dot{IKK_{i}}=k_{i}IKK_{a}-k_{p}IKK_{i}\frac{k_{\mathrm{A20}}}{k_{\mathrm{A20}}+\left[A20\right]\cdot TNF}$$ (13)

$$IKK_{n}={\left[IKK\right]}_{\mathrm{tot}}-IKK_{a}-IKK_{i}$$ (14)

$$TNF=0.5+A\sin\left(\frac{2\pi }{T}t\right)$$ (15)

Here, *N*_*n*_ is the nuclear NF-*κ*B concentration, *I*_*m*_ is the IkB mRNA level, and *I* is the concentration of cytoplasmic I-*κ*B protein.

All the parameters used in the NF-*κ*B model are found in the table below.

We outline briefly the biological correspondences of the different terms in the model:

• In the equation for $\dot{N_{n}}$ , the first term models the import of NF-*κ*B into the nucleus, which is inhibited by NF-*κ*B-I*κ*B complexes formed in the cytoplasm. The second term models the formation of these complexes in the nucleus followed by their export into the cytoplasm.
• The equation for *I*_*RNA*_ describes the NF-*κ*B activated transcription of I*κ*B *m*_*RNA*_ and the spontaneous degradation of the *m*_*RNA*_ with a half-life of ln(2)/*γ*_*m*_.
• The first term in the equation for I*κ*B models translation of I*κ*B *m*_*RNA*_ into I*κ*B protein in the cytoplasm, and the second term models the TNF-triggered degradation of I*κ*B in the cytoplasm when it is bound to NF-*κ*B.
• The triggering stimulus TNF, acts by changing the level of active I*κ*B kinase, [*IKK*_*a*_], which phosphorylates I*κ*B, resulting eventually in its degradation. This degradation rate is set by the parameter α in the model. It is thus only this protein complex with *IKK* that can phosphorylate the NF-*κ*B - I-*κ*B complex and make NF-*κ*B active again.

This model assumes that there is a constant amount of *IKK* (*IKK*_*tot*_), which can be in three states: active (*IKK*_*a*_), inactive(*IKK*_*i*_) and neutral (*IKK*_*tot*_ − *IKK*_*a*_ − *IKK*_*i*_). TNF increases the rate at which neutral *IKK* is made active, and decreases the rate at which inactive *IKK* is made neutral.

## Data Availability

The datasets presented in this study can be found in online repositories. The names of the repository/repositories and accession number(s) can be found below: https://github.com/Mathiasheltberg/TemperaturePrpject. Note that the original videos are too large to be placed in this folder and are available upon request to heltberg@nbi.ku.dk.
